# Veterinary medicinal product regulation in sub-Saharan Africa: identifying barriers and opportunities for enhancing VMP regulatory systems

**DOI:** 10.3389/fvets.2025.1532098

**Published:** 2025-05-09

**Authors:** Alison Z. Pyatt, Suzanne Eckford, Noel Joseph, S. Peter Borriello, Osi Oyati

**Affiliations:** ^1^International Office, Veterinary Medicines Directorate, Department for the Environment, Food and Rural Affairs, Surrey, United Kingdom; ^2^Safe Medicines for Animals-Regulatory Training, London, United Kingdom

**Keywords:** veterinary medicine, regulation, Africa, animal health systems, antimicrobial resistance, pharmaceuticals

## Abstract

Effective regulation of veterinary medicines is essential to ensure veterinarians and animal keepers have access to assured quality, safe and effective products to prevent and treat animal disease. The maturity of national veterinary medicine regulatory systems varies between countries across sub–Saharan Africa and immature systems disincentivise manufacturers from bringing products to these markets. Common barriers to regulatory system strengthening identified by national regulatory agencies (NRAs) in the region include lack of financial resources, lack of trained personnel, and a need for suitable IT platforms to enable work-sharing and sharing of confidential data. Greater convergence and harmonisation of regulatory systems would enable more efficient use of resources through facilitation of regional NRA collaborations. Development of internationally agreed standards and guidance on good regulatory practice, a global regulators forum for exchange of best practice, and application of a self-assessment or audit tool, all of which exist for human medicines regulation, would enable NRAs for veterinary medicines to create and implement institutional development plans to achieve system strengthening. Independent assurance of NRA maturity would enhance opportunity for inter-agency reliance or unilateral recognition of regulatory decisions on product authorisation and good manufacturing practice inspection, currently under-utilised pragmatic approaches to ensuring necessary medicines are available quickly.

## 1 Introduction

Worldwide, livestock farming sectors contribute significantly to food security and trade with an estimated 1.3–1.7 billion people reliant on the sector for their livelihoods, of which approximately 930 million are specified as low-income Africans and South Asians (1, 2). Animal disease continues to present as a major constraint to farming efficiency across these regions, and poor animal health is associated with weak productivity (3). Veterinary medicines are critically important to global animal health, welfare, and productivity, irrespective of the country or region. Veterinarians, veterinary paraprofessionals (VPPs), and livestock farmers need an assured supply chain to permit the necessary access to affordable, safe, good quality and efficacious veterinary medicines required for animal and associated public health. Poor access to VMPs has long been postulated as a causative factor for low agricultural productivity and growth, sustained challenge to animal health, and reduced food security (4).

Weak medicines regulatory capacity, poor access to registered outlets and cost of medical products along with complex manufacturing and trading are some of many potential drivers of the use of poor quality or sub-standard medicines for both humans and animals (5). The use of poor quality or substandard and falsified medicines (SFM) exacerbates production challenges faced by small scale livestock farmers across Sub-Saharan Africa (SSA), increases the risks of antimicrobial resistance (AMR) development (5) and may compound disease risks associated with human consumption of animal products (6, 7). Additional to the risks associated with zoonotic transfer of disease through the consumption of animal products, pharmacologically active substances, including antimicrobials, in the veterinary medicines given to livestock may, if poorly regulated, leave unsafe residues within human food (8). AMR is an ongoing and critical concern worldwide. However, due to poor health care, sanitation, and malnutrition experienced by many in Low-and Middle-Income Countries (LMICs) it is those populations which will be most heavily affected. In livestock farming, poor regulatory compliance and inadequate policy regarding antibiotic usage heightens the risk of AMR within SSA (9). Additionally, the enduring challenge of SFM and associated sub-optimal antibiotic concentration within these products are indicated as a potential contributing factor in emergence and spread of AMR (5).

Urbanisation, population growth, and prosperity across Africa contribute to the observed increase in demand for meat and milk production (10–13). This in turn heightens risk factors for infectious diseases through zoonotic transfer, with the indicated growing markets for meat production also cited to increase epidemic risk (11). In contrast to these risks livestock farming has a noteworthy socioeconomic role in SSA (14). The livestock industry is a significant contributor to the African economy, accounting for between 20 and 50% of the Gross Domestic Product (GDP) for individual countries (15) with many nations heavily reliant upon the sector (16). Livestock farming provides income and employment for many, particularly smallholder farmers in rural areas who make up the majority of farmers in SSA (17), where livestock farming is steeped in longstanding cultural practices and has important social value (15, 18). Across SSA, many smallholder farmers are solely dependent upon agriculture for their livelihoods (19) and investments in agricultural infrastructure and access to markets have been shown to boost incomes for rural communities (20), contributing to poverty reduction, economic growth promotion and overall development in rural areas (21).

### 1.1 Role of national regulatory authorities

NRAs are typically mandated to ensure the quality, safety and efficacy of medicines and correspondingly function to regulate the supply chain of products to the end user (22). For VMPs, these regulatory functions cover manufacture, authorisation, product release (vaccines), and post- authorisation activities including distribution and supply, pharmacovigilance and surveillance of medicine residues in food (23). This provides assurance to veterinarians, VPPs, farmers and animal product consumers on the appropriateness and quality of the products available on the national market, which encourages farmers to invest in VMPs (24). Efficient regulatory processes allow manufacturers to bring their products to market in a predictable and timely manner and provide a level playing field by ensuring adherence to common standards applied across the market. This increases companies’ confidence that their investment in bringing products to market will be protected, thereby encouraging a healthy and competitive marketplace.

NRAs can access guidance from several international organisations. The World Health Organisation (WHO) ‘Good Regulatory Practices’ document sets out the principles and enablers of good regulatory practice for human medicines and the components of a ‘regulatory system’. The regulatory system encompasses the combination of institutions, processes, regulatory framework (incorporating laws, regulations, guidelines and guidance) and resources which, taken together, are integral to effective regulation of medical products in a country or multi country jurisdiction (22), much of which is applicable to veterinary medicines NRAs.

Specific to VMPs, the World Organisation for Animal Health (WOAH) develops international standards, guidelines, and recommendations for veterinary medicines and vaccines. These comprise the requirement for countries to regulate all VMPs, an outline of the core principles to include in national veterinary legislation to assure their quality, safety, and efficacy (25), and detail on the principles of veterinary vaccine production (26). WOAH operates the Veterinary Legislation Support Programme (VLSP), established in 2008 (27) to help countries recognise and address their needs for modern, comprehensive veterinary legislation. Further, there is the Food and Agriculture Organisation (FAO) guidance on national legislation requirements for effective regulation of antimicrobials in the food and agriculture sector (28).

The international standards initiative for harmonisation of the technical requirements for registration of VMPs, VICH, develops non- mandatory guidelines that many countries adopt. Launched in 1996 as a trilateral joint regulator and industry platform between the United States, the European Union (EU) and Japan, with WOAH as observer; membership now includes Canada, New Zealand, Australia, South Africa and the United Kingdom (UK). A parallel initiative, the VICH Forum, was established in 2011 to raise awareness of VICH and VICH guidelines with non-VICH countries (29).

### 1.2 Positioning of national regulatory authorities

**Figure 1:**
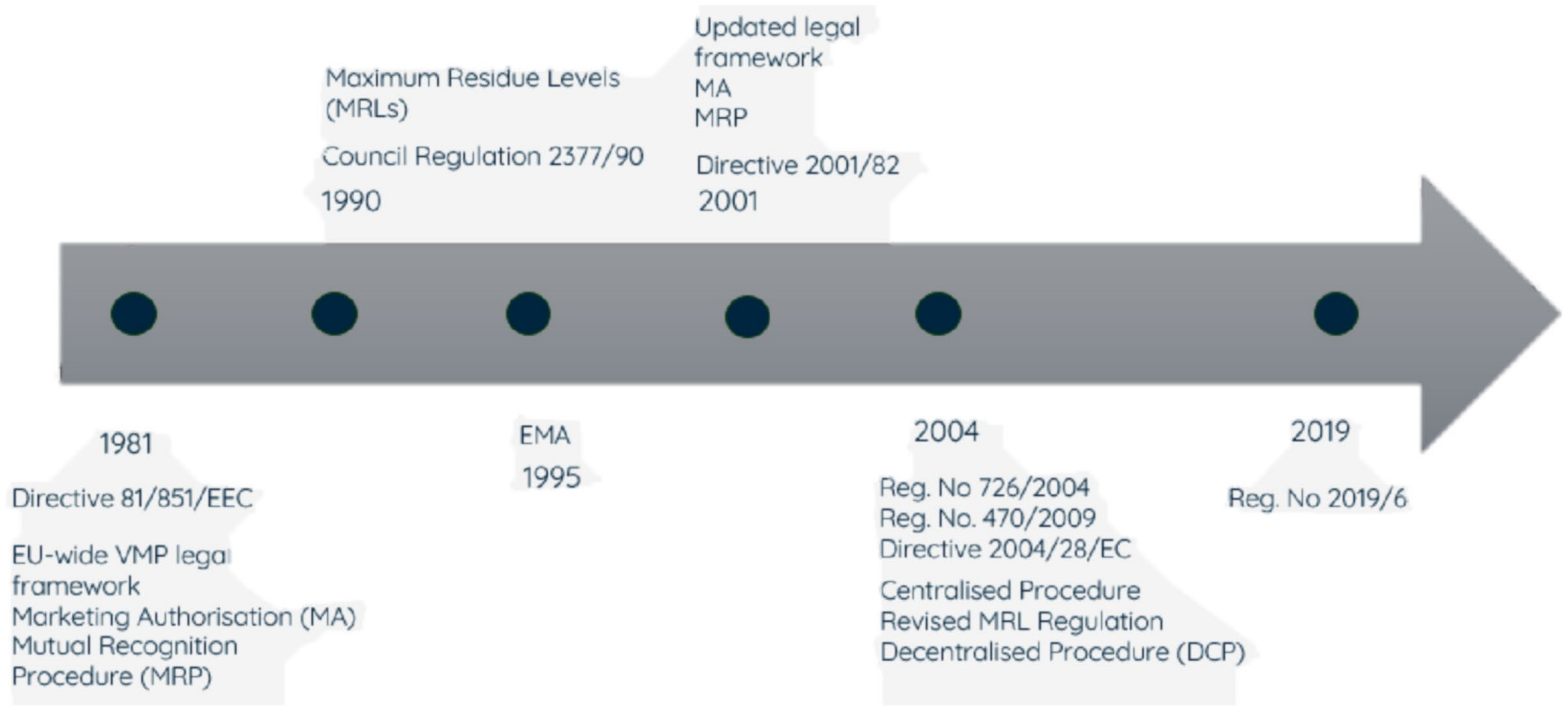
Evolution of veterinary medicinal products (VMP) regulation in the EU.

The governance of national veterinary medicine regulatory systems varies by country; in some cases, there is a national regulatory agency responsible for all aspects of the system. The agency may be veterinary medicine specific or cover both human and veterinary medicines, and in some cases the regulatory mandate may be split across different government departments. The UK Veterinary Medicines Directorate (VMD) is an example of a standalone regulator, though prior to 1989 responsibility was shared within the Ministry of Agriculture, Fisheries and Food. Regulation of VMPs in the EU likewise evolved over many years. The legislation in the different member countries was initially harmonised through European Directives and institutions established to coordinate, and in certain cases centralise, some of the regulatory activities, culminating in 1995 with the establishment of the European Medicines Agency (EMA), (Figure 1). Established to harmonise the work of existing national medicine regulatory bodies, the EMA now coordinates and, in some cases, manages the evaluation and supervision of both human and veterinary pharmaceutical products. There are a variety of routes to authorisation. In addition to country specific national market authorisations there are collaborative routes coordinated by the EMA. The centralised authorisation procedure (CAP) is undertaken by the EMA drawing on a network of experts from over 40 NRAs of EU Member States (MSs), and medicines authorised by this route can then be marketed in all MSs. The decentralised authorisation procedure (DCP) is where the ensuing authorised product is simultaneously valid in several member states, as selected by the manufacturer, with scientific assessment conducted by a nominated lead and reviewed by the other involved MSs. A manufacturer can apply to have an existing national authorisation mutually recognised simultaneously (‘MRP’) by additional MSs. Legislation also permits the subsequent recognition (SRP) of a product authorised by the MRP or DCP routes.

### 1.3 Supply chain for veterinary medicinal products

**Figure 2:**
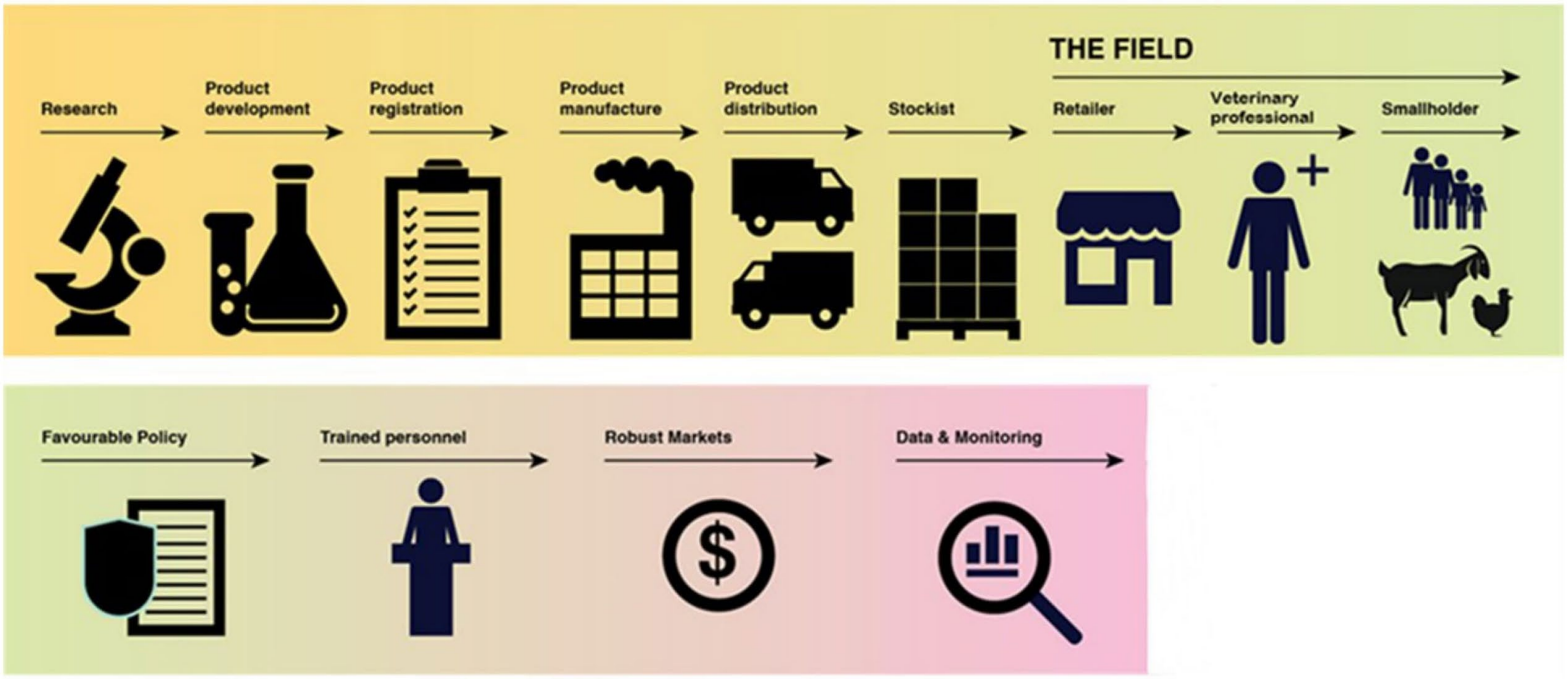
The supply chain for end -to-end veterinary medicinal product lifecycle. Adapted and reproduced with permission from GALVmed, 2023.

Effective regulation is only one element of enabling the medicines supply chain. The process of bringing a VMP to market is complex; requiring contributions from a wide range of organisations and actors and is a process which may vary according to regional and national legislation. Irrespective of this complexity, for improved availability of good quality VMPs the livestock sector is inherently reliant upon the functionality and successful interconnectivity of the supply chain. There are a multitude of aspects requiring consideration to achieve the end goal of enhanced VMP availability (Figure 2). Furthermore, externalities, such as distribution infrastructure and the availability of veterinary technical expertise, can also impact access to veterinary services and VMP availability (30, 31). As illustrated in Figure 2 the process commences with research to understand market needs, which is followed by investment into product discovery, development, and clinical trials, ultimately leading to product registration and authorisation, scaled manufacture, distribution and consequently product availability to the end-user.

This policy and practice review evaluates the role of regulators in this complex supply chain and seeks to understand how regulatory enhancement can support the development of an enabling environment through mechanisms such as harmonisation of processes and regulator-to-regulator support.

### 1.4 Veterinary medicines regulatory systems in sub-Saharan Africa

Veterinary medicines regulatory frameworks in SSA countries are varied and often insufficient, failing to ensure access to high-quality medicines for livestock keepers (32). Furthermore, SSA NRAs have different organisational forms. Some countries have established veterinary medicines specific agencies, for example the Veterinary Drug and Animal Feed Administration and Control Authority (VDFACA) in Ethiopia (32, 33) while others have merged their human and veterinary medicines regulatory functions to form a joint agency, as in Uganda (34, 35). Some countries have divisions and departments within the Ministry of Agriculture or Livestock that retain the regulatory mandate, for example Cameroon (36), while in South Africa, the role of regulating veterinary medicines falls between its joint human and veterinary South African Health Products Regulatory Authority (SAHPRA) and the now termed Department of Agriculture, Land Reform and Rural Development (DALRRD) (37, 38).

Some formal or informal collaboration between national regulators exists in the West, East and Southern Africa regions. In West Africa, the eight countries forming the West African Economic and Monetary Union (WAEMU) established a regional structure for a centralised authorisation procedure (CAP) of VMPs in 2006, which has been operational since 2010 (39). In contrast, the East Africa Community (EAC) developed a harmonised registration system, initially for veterinary biological products, based on a mutual recognition process (MRP) (39). This MRP is based on one NRA leading the assessment of a product application while others contribute. After reaching a consensus on the scientific evaluation, a non-binding recommendation for approval or refusal is issued by the assessment team, based on which each participating country should grant a national product authorisation (40). The Southern Africa Development Community (SADC) has adopted Regional Guidelines for the Regulation of Veterinary Drugs in Southern Africa (41).

To be effective, regulation must be underpinned by an appropriate legislative framework. Some countries, such as Mozambique, lack specific veterinary drug legislation (42) and in others, such as Angola, veterinary drugs are minimally covered in legislation focused on human medicines (43). In Kenya, Malawi, Tanzania, and Uganda respectively, veterinary pharmaceuticals were regulated by Pharmacy Boards under the Ministry of Health (36). In countries where the Ministry of Health oversees veterinary pharmaceuticals, veterinary professionals are underrepresented, and animal health product registration is seldom prioritised (39). Dispensing of these products is strictly regulated by pharmacists, prohibiting veterinary surgeons from distributing medicines without a registered pharmacist (39). Veterinary Boards in Kenya, Uganda, and Tanzania sought legal authority to fully regulate veterinary drugs (39) and all have since secured legal powers to do so, albeit relatively recently. The Kenya Veterinary Medicines Directorate was launched in 2017 after being established in 2015 under the Veterinary Surgeons and Veterinary Paraprofessionals (VSVP) Act CAP 366 (44). Joint human and veterinary agencies were established in both Uganda (35) and then Tanzania (34, 45).

Existing legislation is often ineffective and varies widely, which hinders a unified regional market within the SSA animal health sector (32). Although the majority of African countries have registration systems or related legislation in place, many do not effectively regulate VMP use in the country (39). In some countries the veterinary medicines supply chain operates informally and without regulation (46, 47). Similar issues of inadequate legislation, shortage of skilled regulatory experts, and poor regional partnerships among NRAs also negatively impact on availability of human medicines (48, 49). The African Medicines Regulatory Harmonisation (AMRH) initiative aims to address these obstacles by streamlining the approval process for human medical products and aligning national laws with the African Union Model Law on Medical Products Regulation (48).

### 1.5 Assessment of VMP regulations and the implications of harmonisation

The issues of under-resourcing of veterinary services (50), and poor access to animal health professionals and veterinary health systems for rural farming communities in SSA are well documented (51, 52). Conversely, limitations in VMP access due to regulatory process are much less understood and with limited research evaluating the role of policy and concurrent practice in enhanced SSA farmer access to VMPs.

Immature authorisation and post authorisation regulatory processes serve as a disincentive to the pharmaceutical industry, as prevalence of low-cost illegal alternatives discourages market investment by legitimate companies, and to veterinarians, VPPs, and farmers to invest in using products, as they have no assurance of medicine quality or effectiveness. More widely, fragmented markets and inadequate and uncoordinated regulatory controls allow substandard and falsified products to circulate particularly in regions where there is regulatory divergence in bordering countries (53). Manufacturers producing medicines to accepted international standards avoid VMP markets in SSA as the regulatory framework is neither transparent nor predictable. This deprives veterinarians and farmers of choice and limits access to veterinary medicines.

A strong, harmonised VMP regulatory framework can be considered as critical infrastructure for animal health and therefore for livestock productivity (54). A review of existing co-operative initiatives to identify factors that contributed to success (32) explored the origin, structure, resourcing, legal and political aspects, and infrastructure requirements of eight harmonisation initiatives in different sectors; the EU, SADC, EAC, WAEMU, VICH, the Association of Southeast Asian Nations (ASEAN), the New Partnerships for Africa’s Development (NEPAD), and the World Trade Organisation (WTO) agreement on the application of sanitary and phytosanitary measures (SPS). This identified a number of key elements that supported success for harmonisation/convergence efforts which could be applied to initiatives to improve VMP regulation harmonisation initiatives (32).

## 2 Review aims and objectives

This review focuses on the status of implementation of VMP regulation in SSA, including organisational structures, operational processes, and existing collaborative initiatives between SSA NRAs. It draws extensively, but not exclusively, on prior research (32) which collated and analysed detailed evidence on 28 SSA countries and from a broad range of stakeholders. This evidence base has been enhanced through further desk-based research into regulatory tools and frameworks and through qualitative stakeholder feedback gathered through a series of regional workshops. The review also seeks to explore the barriers to implementation of effective and efficient VMP regulation, and to identify improvement opportunities for countries, including through greater mobilisation of harmonised approaches to VMP authorisation, and further development of enabling tools.

For clarity the following definitions are adopted. The term *registration* includes the process of assessing application data packages (known as dossiers) and issuance of marketing authorisation or product licences. The term *regulatory harmonisation* represents a process where regulatory authorities align technical requirements and guidelines for authorisation and post-authorisation activities related to VMPs. The term *reliance* is the act whereby the regulatory authority in one jurisdiction considers and gives significant weight to assessments performed by another NRA or trusted institution, or to any other authoritative information, in reaching its own decision. The term *work sharing* refers to collaborative processes where multiple NRAs work together by dividing the application dossier between them to evaluate VMPs.

## 3 Methodological approach

Prior research (32) gathered detailed evidence from 28 SSA countries. A mixed methods approach was applied using a triangulation technique incorporating scoping literature review, quantitative surveying utilising closed and open questioning techniques to encourage expansive answers, and qualitative interviews. Typical literature sources reviewed included primary information such as legislation and policy documentation, and secondary information comprised of reports commissioned by international organisations such as WOAH and the World Bank. Questionnaires (42 respondents) were used to capture and validate key legislative and regulatory information from the perspective of an NRA or national officials and other relevant stakeholders, such as WOAH and FAO. Follow-up qualitative interviews were conducted with nine selected questionnaire respondents, and email correspondence conducted with the other participants to clarify discrepancies, fill gaps and to draw on their views and understanding of the regulatory landscape. Case studies explored in detail the operationalisation of eight harmonisation initiatives. Data collected from primary research, case studies, surveying, and interviews, were evaluated to perform a gap analysis of regulatory standards and systems relating to the registration of VMPs within the 28 target countries, and to assess factors influencing regulatory harmonisation.

A desk review was conducted to identify tools that would enable VMP regulation improvement and harmonisation. Four existing tools were identified with relevance to medicines regulation: the WHO Global Benchmarking Tool (GBT) for human medicine NRAs; WOAH’s Performance of Veterinary Services (PVS) Pathway tool (50); the European Heads of Medicines Agencies (HMA) Benchmarking of European Medicines Agencies (BEMA); and the World Bank’s Enabling the Business of Agriculture (EBA) reports. A comparison of the scope of these tools was undertaken to ascertain suitability for adaptation to global application in the veterinary medicines regulation sector.

Stakeholder engagement was undertaken with 41 countries and Regional Economic Communities (RECs) through a series of virtual and physical meetings. In addition, regional workshops were conducted with national, regional, and international stakeholders in South Africa, Cameroon, Tanzania, and Cote d’Ivoire between July 2020 and December 2022, convening regulators, RECs and other actors to engage in dialogue, exchange insights and foster collaborative approaches. The workshops reviewed themes identified through the previous research and aimed to bridge gaps, promote transparency and to develop recommendations on how to improve and strengthen regulatory frameworks. In some regions, this was the first such gathering providing the opportunity to discuss the challenges in each region and explore potential solutions.

To the best of the authors’ knowledge the detail here provided is accurate at the time of writing.

## 4 Outcomes and discussion

We present and highlight here results concerning regulatory systems and structures, policy tools, and perspectives on resource and operational factors which, through this course of this inquiry, were indicated as key factors affecting availability of and access to VMPs across SSA. Subsequently, we indicate potential opportunities for regional enhancement through the adoption of harmonised approaches and outline salient recommendations.

### 4.1 Institutional structure and capacity

**Figure 3:**
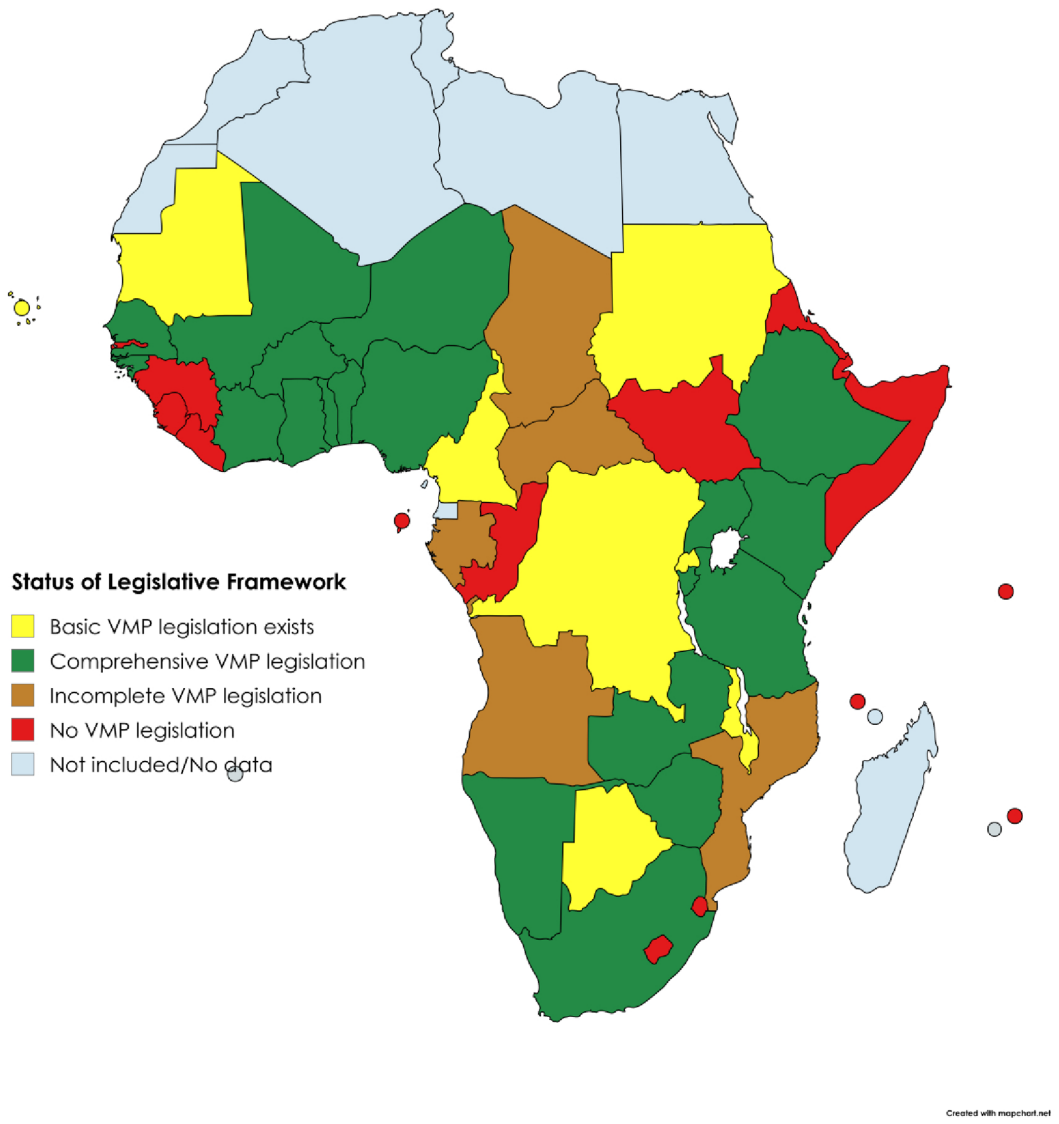
Status of VMP legislative framework in SSA.

**Figure 4:**
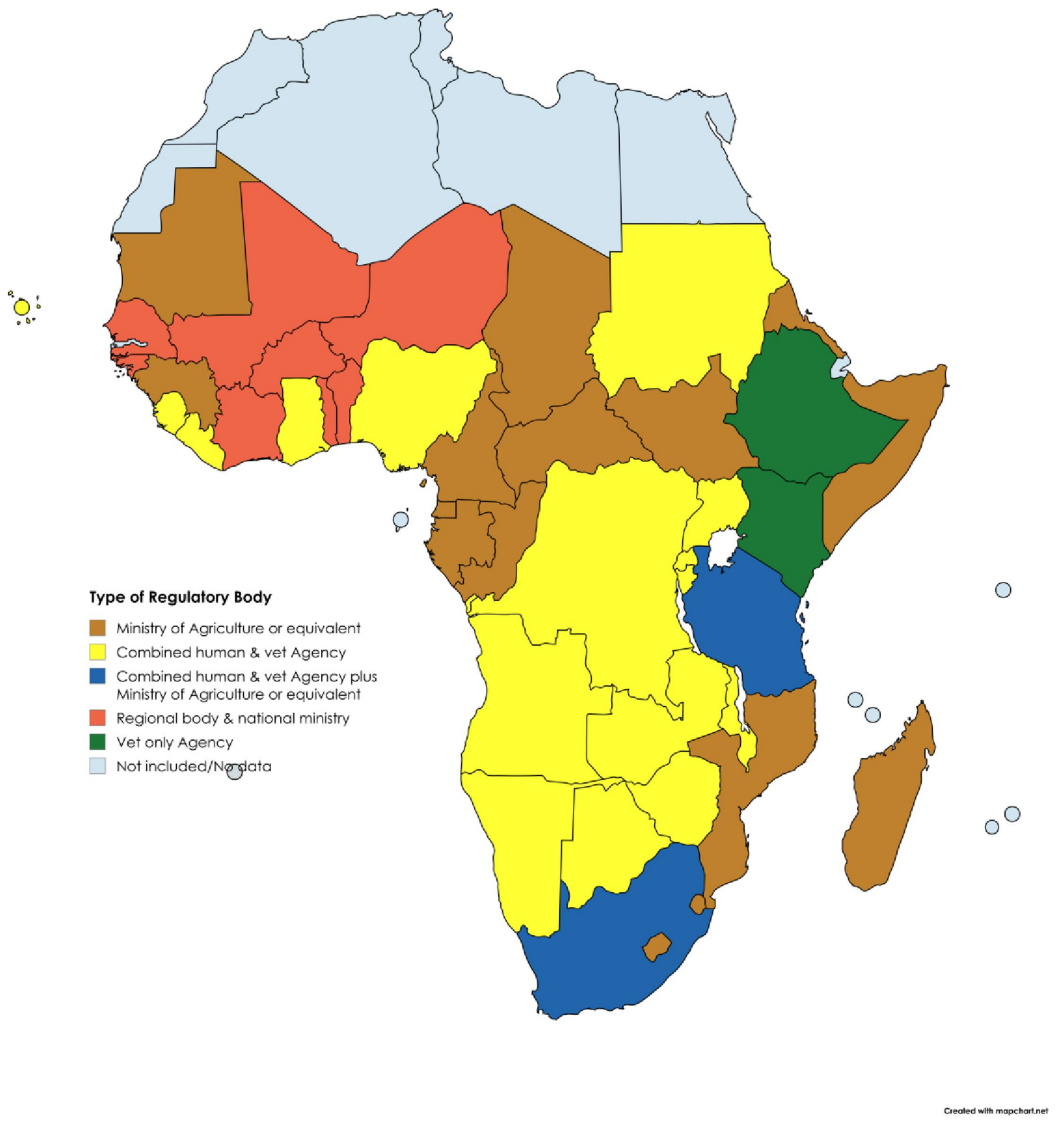
Institutional setup for VMP regulation in SSA.

Key to understanding the status and future potential of VMP regulation was the development of a holistic overview of NRA characteristics within each region of SSA (Tables 1, 2) (32). Organisational setup of the responsible authorities and status of the regulatory framework for VMPs varies between regions and between countries within each region, as also shown in Figures 3, 4 (32).

**Table 1:** Overview of NRA regional characteristics [Source: (32)].

Region	Countries	Characteristics
Southern Africa	Angola Botswana Malawi Mozambique Namibia South Africa Zambia Zimbabwe	Countries appear to have varying capacity to effectively regulate VMPs. Mozambique - there is no legislation in place for addressing VMPs. Angola VMPs are only superficially mentioned in legislation, which is targeted to human medicines. Restructuring of regulatory processes in South Africa and Botswana to improve regulatory activities. Botswana and Malawi are actively working to improve and update their legislative frameworks and align with international regulatory standards. There is VMP regulation that allows the countries to regulate VMPs with accompanying guidelines (Botswana, Malawi, Namibia, South Africa, Zambia, and Zimbabwe).
Eastern Africa	Eritrea Ethiopia Kenya Rwanda South Sudan, Sudan, Tanzania Uganda	There is evidence of good VMP regulation in some of these countries and a degree of cooperation to regulate VMPs at the regional level (Mutual recognition procedure; MRP). A few countries had no evidence of a system to regulate VMPs (Eritrea and South Sudan). There are varying levels of VMP regulation within the region. There is an EAC MRP in which members (Kenya, Tanzania and Uganda) have a good legislative framework and functioning regulatory systems. These include the presence of a comprehensive legislation supported by clear and accessible guidelines for applicants which have been aided by harmonisation efforts in the region. Rwanda has since caught up and has become an active participant in the EAC MRP. Ethiopia and Sudan also have some regulatory capacity and legislative frameworks in place specifically for VMPs. Ethiopia has established a regulatory body for VMPs within a newly created authority and Sudan has a joint human and veterinary agency. Although there are countries within the region that have clear regulatory structures in place (Kenya and Uganda), the challenges of insufficient resources and have impacted the registration process.
Western Africa	WAEMU: Burkina Faso Côte d’Ivoire Mali Niger Non WAEMU: Ghana Mauritania Nigeria	WAEMU members, which are all French speaking countries, utilise a centralised system for VMP registration. WAEMU has a centralised system for the registration for VMPs and issues MAs through a Regional Committee for VMPs (CRMV) which is valid in all eight member countries. Countries within WAEMU have successfully harmonised and adopted WAEMU legislation into their national legislative frameworks for regulating VMPs. Mauritania, although a French speaking country, is not a member of WAEMU. Ghana, Mauritania, and Nigeria are not part of any regional harmonisation initiative for VMP regulation. These countries generally have good legislative frameworks and institutional capacity to implement and enforce regulatory standards.
Central Africa	Cameroon Chad Central African Republic Gabon Congo DRC	Legislative frameworks within the region are inadequate and lack the institutional capacity to implement and enforce regulatory standards Cameroon and DRC were identified to have a legislative framework establishing regulatory standards and structures for VMPs.

**Not all countries in a region are shown-table contents are restricted to those reviewed in 2019.

**Table 2:** Key characteristics of VMP regulation status and activity.

Region / Country	Legislative framework	Regional cooperation	Product registrations	Online submission
Eastern Africa				
Kenya (V)^1,2^, Rwanda (J), Tanzania (J)*^2^, Uganda (J)^2^	+	+	+	+
Burundi (J)	+	+	i	−
Ethiopia (V)^2^, Sudan (J)^2^	+	−	+	−
Eritrea (MA)^2^, South Sudan (MA)^2^	−	−	−	−
Djibouti (–––), Somalia (MA)	−	−	−	−
Southern Africa				
Botswana (J)^2^, South Africa (J)*^2^, Zambia (J)^2^	+	+	+	+
Zimbabwe (J)^2^, Namibia (J)^2^	+	+	+	−
Malawi (J)^2^	+	#	+	−
Angola (J)^2^, Mozambique (MA)^2^	+	−	+	−
Madagascar (MA)	+	−	−	−
Comoros (−), Eswatini (MA), Lesotho (MA)	−	−	−	−
Seychelles (−), Mauritius (−)				
Western Africa				
Benin (R), Burkina Faso (R)^2^, Cote d’Ivoire (R)^2^, Guinea-Bissau (R), Mali (R)^2^, Niger (R)^2^, Senegal (R), Togo (R)	+	+	+	i
Ghana (J)^2^, Nigeria (J)^2^	+	−	+	+
Cape Verde (J)	−	−	+	−
Mauritania (MA)^2^	+	−	−	−
Guinea (MA), Liberia (J), Sierra Leone (J), The Gambia (−)	−	−	−	−
Central Africa				
Democratic Republic of Congo (J)^2^	+	+	+	−
Cameroon (MA)^2^, Chad (MA)^2^, Central African Republic (MA)^2^, Gabon (MA)^2^	+	−	+	−
Congo (MA)^2^, Sao Tome and Principe (−)	−	−	−	−

(..)^1^: The letter in parentheses denotes the type of regulatory body; (V): Standalone (vet only). (J): Joint Agency (human & vet); (MA): Ministry of Agriculture; (R): Regional body (West Africa Economic & Monetary Union—WAEMU); *Two bodies share responsibility for VMP’s; i, Being established; #,Observer; + / – / –––: Present / Absent / Unknown. (..)^2^: Detailed individual country synopses (32). Source: (32, 59).

The different organisational forms correlate with varying levels of NRA effectiveness, confirming the findings of previous studies (36). Those with a specific mandate for veterinary medicines typically demonstrate better levels of functionality, whether centralised regionally, or retained nationally as a veterinary only or combined human and veterinary agency. In contrast, countries lacking a dedicated regulatory body, where responsibility falls on units or departments within broader Ministries of Agriculture or Livestock (for example as seen in Mozambique, Congo, and South Sudan), often have shortcomings.

However, interviews with representatives of NRAs in the region indicated that joint agencies also have shortcomings, with veterinary medicine regulation sometimes falling behind that of human medicine oversight in countries such as Angola, Liberia, and Sierra Leone. While there are commonalities between regulation of human and veterinary medicines which, in theory, should lead to greater efficiency when regulating under a joint mandate, a common theme in joint agencies is the significant disparity in allocation of financial and human resources to human over veterinary medicines regulation. This reflects wider global disparity in the profile and resource allocation between human and animal health, which fails to take account of the inter-relatedness of these systems, such that failing to effectively regulate veterinary medicines can have a resultant negative impact on public health (5). Detaching the regulation of veterinary medicines from key interested stakeholders, for example bodies responsible for wider animal health systems, veterinary professionals and livestock keepers, interrupts communication feedback loops essential for effective regulation, for example the reporting of adverse events, intelligence on product availability and suitability, and effectiveness of product withdrawal periods.

**Figure 5:**
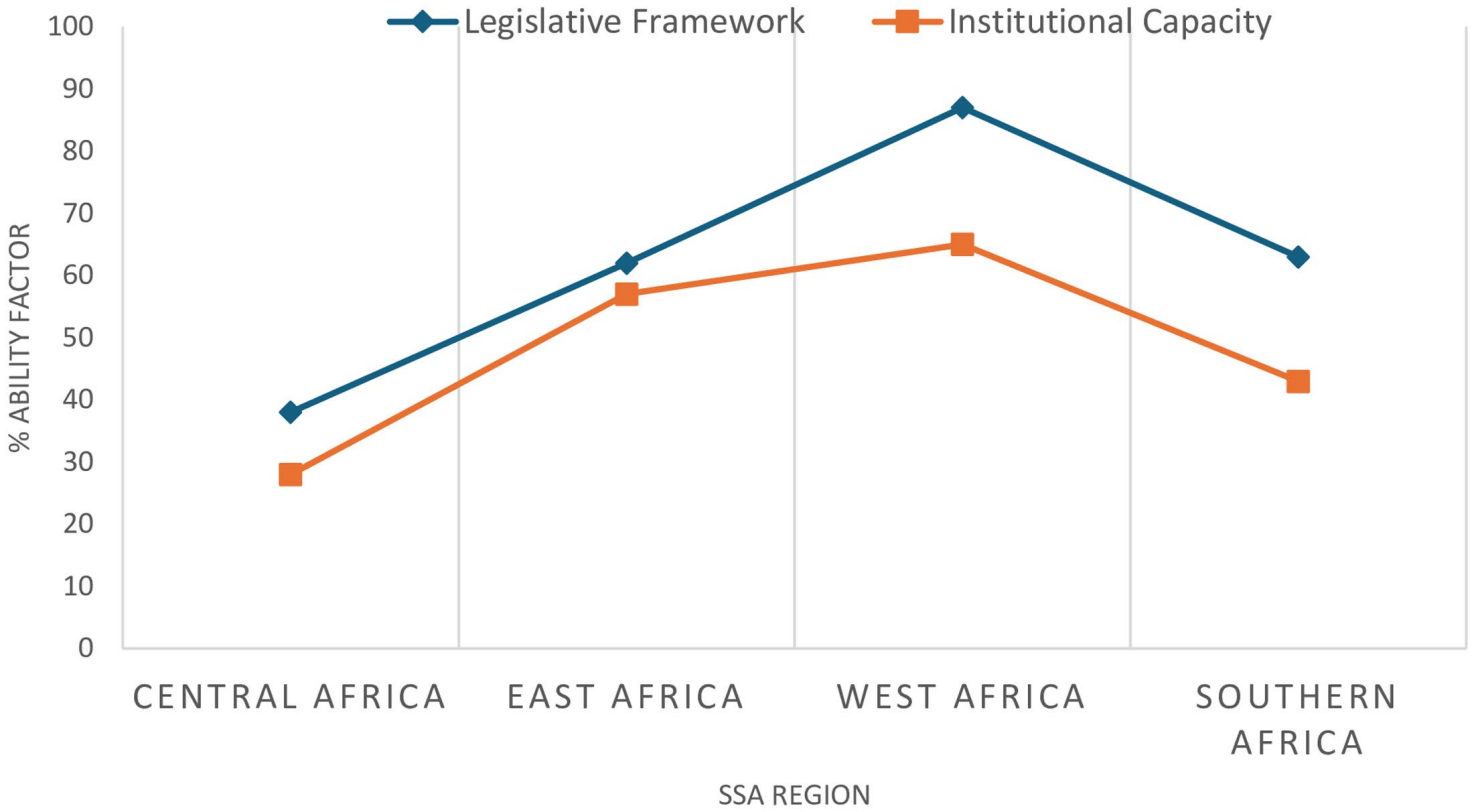
Regional average comparison in legislative framework and institutional capacity scores [published in VMD (32)].

Participation in a regional collaboration also varies between NRAs, with a correlation seen between participation in regional regulatory activities and the presence of product registration process and the ability to accept online applications for product registration, both selected as markers of regulatory effectiveness. This affirms the premise that collaboration benefits NRAs, for example through improved efficiency by avoiding duplication of efforts as achieved through the WAEMU centralised process, or by improved communication and information sharing between the countries participating in the EAC MRP. Four key ‘ability factors’ have been proposed to determine a country’s readiness to participate in regional collaboration (32), derived by using a modified PESTEL (Political, Economic, Social, Technological, Environmental and Legal) approach to analyse drivers of change in a strategic environment; these ‘ability factors’ are the national legislative framework, institutional capacity, political environment and economic situation (Table 3). Data collected regarding countries’ ability factors was analysed to determine a ‘Readiness Score’ which represented the likelihood of a country achieving regulatory harmonisation. Figure 5 (32) shows regional averages of the Readiness Scores assigned to countries. The average score was highest in West Africa because four of the countries examined within that region were participants in the WAEMU centralised system of regulation. As indicated by the average scores in Central Africa, countries in that region have lower institutional capacity to implement and enforce the limited legislation in place. Although the average scores of other regions are higher, challenges in institutional capacity and the comprehensiveness of the legislative frameworks also exist in some countries within those regions.

**Table 3:** Ability factors.

Ability factor	Description
Legislative framework	Legislative framework for VMP regulatory standards Supporting regulatory structures in place to implement and enforce regulatory standards
Institutional capacity	Ability to implement regulatory standards Governance capacity to support regulatory authorities Institutional technological capacity (IT infrastructure) Institutional sustainability and structural composition
Political environment	Political environment of VMP regulation Ability of political actors to implement VMP regulatory standards
Economic situation	Economic drivers directly affecting internal and external investments into livestock health

### 4.2 Regional collaborations

As indicated, regional engagement activities were fundamental to the project and the appraisal of VMP regulation in SSA. Participants of the four regional workshops articulated the need to advance existing regional collaborative structures and processes to realise the potential benefits in increased efficiency in a resource constrained landscape. RECs are crucial for advancing harmonisation initiatives, and the effectiveness of harmonisation efforts relies on the political support and resources of the RECs. Their responsibilities include setting up appropriate regulatory frameworks and coordinating regional activities. The legal foundation of the RECs and their ability to effectively enforce the implementation of regional regulation impacts outcome success. For example, SADC has the authority to establish regional policies and protocols, but it does not possess legislative power to enact laws directly applicable in all member countries, whereas the Economic Community of West African States (ECOWAS) has the mandate to enact binding regulations for its member states.

In Central Africa, workshop participants identified that development of a functional regional system would require five additional enabling Regulations and directives to be adopted by the Economic and Monetary Community of Central Africa (CEMAC) to build on the existing 2017 Community Regulation No. 09/17 (55) establishing the legal basis for a centralised authorisation system in the region, akin to that already operational in WAEMU. In West Africa, despite ECOWAS introducing regulation in 2010 that established the mandate for a region-wide centralised system and the establishment of a regional veterinary committee, awareness of this legislation remained poor. This lack of understanding and awareness of existing regulations was previously highlighted in a 2014 study commissioned by ECOWAS (56), indicating little progress in implementation in the intervening period. The establishment by ECOWAS of the Regional Animal Health Centre (ECOWAS-RAHC), operational since 2018, was intended to enhance regional harmonisation and fulfil the role of stimulating the initiative. Unfortunately, a lack of enforcement of the regulation, lack of human and financial resources and a reluctance by member countries to engage due to perceived lack of benefit, have acted as barriers to progression. However, a recent meeting of the ECOWAS regional veterinary committee to discuss the implementation of the ECOWAS regulations on VMPs, indicated a desire to progress (57). Across both Central and West Africa there emerged a common theme indicating the need for greater sensitisation of member states into the regional vision. While the immediate benefit of a regional approach might be greater for those countries with immature regulatory systems, ultimately a regional system benefits all countries. On a practical basis the regional bodies should attempt to develop functional networks of focal points from the departments or agencies responsible for VMP regulation within member countries, although it is noted that identifying appropriate contact points is difficult, a difficulty not restricted to SSA. In East and Southern Africa, the regional initiatives focus on collaboration between NRAs rather than a centralised system. Although East Africa is more advanced in establishing their procedure, with the GALVmed supported EAC MRP, which started with vaccines but now expanded to pharmaceuticals, with a number of products authorised by this route, only Kenya, Tanzania, Uganda and Rwanda are actively participating. Burundi, South Sudan and, more recently DRC, act as observers since they have not yet established fully operational NRAs (32). Although Burundi has since established the Burundian Regulatory Authority for Medicines for Human Use and Food (58), the veterinary medicines regulatory functions are not yet fully operational. Expanding the procedure to include other countries in the wider Eastern Africa region with established regulatory bodies and substantive livestock populations, for example, Ethiopia, would potentially make the MRP process more attractive to manufacturers.

Misunderstanding by manufacturers on the MRP procedure, for example that dossiers have to be submitted simultaneously to all NRAs not through the lead NRA, indicates that communication between regulators and industry could be improved. Variable procedure timelines, which are a source of frustration to manufacturers, are likely due to the lack of available resources in the participating NRAs. Exploring new ways of work-sharing could improve the process, such as the one employed by the EAC human medicine MRP which divides the lead (Reference Country) role between NRAs according to different sections of the dossier, whereby one country may lead in the evaluation of one section of the dossier and another one with the remainder. Regular industry-regulator workshops, and additional guidance were also indicated as valuable activities to improve manufacturers’ understanding of the procedure.

Regulatory collaboration in Southern Africa has progressed more slowly than in EAC; this is likely due to the lack of a dedicated resource to coordinate activities. Operationalisation of collaborative authorisation processes is more successful when dedicated resource such as a regional coordinator or coordinating body are employed, as demonstrated in West and East Africa. Despite this absence of external support, Zambia, Zimbabwe, Botswana, Namibia, South Africa and Malawi have developed an initiative termed vet-ZAZIBONA, agreed a collaborative authorisation procedure, developed guidance, sensitised industry, and opened a pilot application phase in 2022. This procedure for VMP harmonisation was presented to the SADC Livestock Committee and then the Council of Ministers in 2023 and has received political endorsement. Although the procedure stalled, due to a lack of available assessor resources in the participating agencies, in part due to staff turnover, and lack of funding to convene the requisite assessor meetings, it has recently resumed.

In all of the regions there is scope for countries that do not yet have fully functional regulatory capacity to unilaterally recognise the regulatory decisions issued by other established regulators; for some countries this may require amendment to their national legislation; in all cases countries would need to have assurance in the processes conducted by the established regulator.

Both Eastern and Southern Africa NRAs highlighted the need for a shared secure IT platform to assist their collaborative procedures; this would allow manufacturers to submit one application to be accessed by all participating NRAs, would allow sharing of secure information between NRAs when conducting assessments, and would reduce duplication and facilitate interagency and industry - regulator communication. Regulators from all of the regions called for access to a comprehensive training programme to build staff capacity and capability, together with a framework against which the assessors’ competency could be evaluated.

Recognition of the significant challenges posed by unauthorised or SFM with potential risks to users, livestock, the environment and AMR development was also clearly indicated. Feedback from workshop contributors underscored the need for robust regulatory systems, quality control and pharmacovigilance to address these challenges effectively. The involvement of industry stakeholders and harmonisation were cited as key factors in combating the circulation of SFM products. In this context, the availability of a published authorised national product list will not only help to strengthen regulatory oversight but can also enhance transparency, aiding in the identification of unauthorised products.

### 4.3 Operational perspectives

Common challenges to the implementation of effective regulation identified at the regional workshops included transparency and access to regulators, and the need for mechanisms to support collaboration and communication across the region between regulators working together through the existing regional initiatives, and between industry and regulators. Participants in the Eastern Africa workshop identified a lack of awareness by medicine manufacturers of the existence of the regional legislation and mandate and concluded that further sensitisation and engagement was needed to increase awareness and secure buy-in for its implementation.

A common barrier to improving engagement and communication was the difficulty in identifying contact details for national government departments or agencies responsible for VMP regulation. This finding was compounded by feedback from industry focal points during interviews, who outlined the disincentives to communication with regulators presented for a company seeking to enter a new market. In order to address the difficulty in identifying the NRAs responsible in the different countries, a data gathering exercise was completed to develop and publish a Global Database of VMP Regulators, available on the Safe Medicines for Animals-regulatory training website (59). This database is a source of contact information for the agencies/institutions responsible for the authorisation of veterinary medicines (pharmaceutical products and vaccines) and GMP inspections in each country. It contains a description of the responsibilities of those involved in the regulation of veterinary medicines within a country, alongside specific veterinary contact points for each of the bodies that discharge those responsibilities. Where there are established regional bodies, alongside national ones involved in the regulation of veterinary medicines, these have been included in each country’s details involved. It is anticipated that the database will benefit regulatory experts working in the different agencies/institutions, industry and other stakeholders by enabling collaboration and the sharing of knowledge on aspects of veterinary medicines regulation.

Successful collaboration between regulators is dependent on inter-agency trust, which is underpinned by communication and by an assurance of adherence to acceptable processes, procedures and standards performed to agreed timelines. Barriers to collaboration identified through the stakeholder interviews and through the workshop discussions included the lack of a secure communication mechanism between regulators for sharing of confidential information, the lack of transparency of national regulatory processes and lack of assurance of the maturity of a regulatory body. In human medicine regulation, the WHO Global Benchmarking Tool is a platform for independent assurance of the stringency of a regulatory body (60). Countries can apply this tool as a self-assessment process to inform their development pathway and can also request WHO to perform an external evaluation and assign a maturity level rating to their national regulator. This maturity rating provides assurance to other countries on the suitability of the regulatory system, processes and decisions, and thus can further be used by countries to identify which regulators’ decisions they may wish to unilaterally recognise. The EMA operates a similar benchmarking exercise for veterinary and human medicines NRAs, but do not make the findings publicly available, and do not assign maturity scores.

Stakeholder engagement highlighted the need for a tool that enables assessment of the level of ‘maturity’ of veterinary NRAs, to drive improvement in agency performance and to facilitate inter-agency collaboration and regulatory harmonisation. Stakeholder consultation on identified existing tools through the regional workshops demonstrated preference for the WHO GBT as the most appropriate format on which to model a veterinary equivalent. Further consultation with the WHO team responsible for the GBT, veterinary medicine regulators and the veterinary pharmaceutical industry identified that a veterinary tool should follow the template of the WHO GBT in order to ensure complementarity and familiarity to joint veterinary and human regulators, while ensuring functionality supports the aspects specific to veterinary medicines regulatory systems. Accordingly, a tool has been developed by the project team, which is currently being piloted.

Communication between agencies is inhibited by the current lack of secure electronic means to share confidential information including a lack of secure email. Many agencies do not operate through secure government platforms or have an electronic platform through which confidential documentation can be shared between agencies involved in collaborative procedures. For some countries there is no capacity to receive licensing or authorisation applications from manufacturers electronically.

Veterinary drug manufacturers (local and international) are generally required to submit an application (dossier) to the relevant regulatory authorities for approval or authorisation before the product can be made available on the market. Although several SSA NRAs now accept dossiers electronically, some are still reliant upon paper-based systems. NRAs need IT infrastructure to allow them to store the electronic dossiers and their evaluation reports securely and correspondingly better collaborate with other NRAs. The need for a functional IT system is therefore a key stakeholder recommendation irrespective of region or sector (regulator or industry).

### 4.4 Resource access

Human, financial, and infrastructure resource insufficiencies were repeatedly evidenced as important themes, as also highlighted by the small number of development programmes in an inventory of recent and current initiatives to improve regulation of VMPs in SSA.

Availability of appropriately skilled and trained technical staff was consistently cited as an ongoing and significant barrier to improving VMP regulation and progression in regulatory harmonisation across SSA. This trend was evident across all SSA regions and was consistently reported from both NRAs and industry. Compounding the skills shortage is a high staff turnover rate with frequent movement from regulatory roles to industry post training, and consequently the need to be continually training new staff, and the inability to draw on a suitably trained pool of staff. The ongoing draw on both financial and time resources associated with repetitious in-house training was also highlighted by regulators as a significant barrier to regulatory improvement. In more detailed discussions regarding education and training (particularly at postgraduate level), access to high quality, consistent and cost-effective training resources and materials were strongly indicated as barriers to ensuring the maintenance of an adequately trained and skilled workforce. The WHO hosts a programme of regulatory system strengthening for human medicine regulators, which encompasses elements including guidance, for example, the Good Regulatory Practice Guidance, frameworks, for example the Global Competency Framework for regulators of medicines, tools, for example. The Global Benchmarking Tool for evaluation of regulatory systems, and networks, for example. The Coalition of Interested Parties (61). These elements provide NRAs with a structured approach to system strengthening, including training and development of their workforce. Having parallel initiatives available for veterinary NRAs would likewise support their development efforts.

Government preparedness or capacity to provide suitable levels of financial support for veterinary regulatory enhancement were indicated as ongoing challenges. These issues are, however, complex to address due to country level idiosyncrasies. Furthermore, in some cases the fees charged to industry for marketing authorisation are bound by legislation and so difficult to change, and in most instances fee payments go directly to the government treasury and not to the regulator. There is an apparent disconnect between the cost of providing the regulatory service and fees charged, which hinders NRAs in utilising finances to shape the market and thus encouraging manufacturers to bring essential medicines to the market.

### 4.5 Strategic approaches to improvement

The SSA veterinary regulatory authorities should seek to establish a collective, sustainable and efficient system for regulating VMPs without compromising drug availability. VMPs need to be regulated in accordance with the global best practices while being cognisant of the importance of availability of these products by reducing unnecessary bottlenecks, for example by focusing more on mutual or unilateral reliance.

An area in VMP regulation where mutual reliance has been successfully implemented is in Good Manufacturing Practice (GMP) inspection among some members of the Co-operation Scheme of the Pharmaceutical Inspection Convention (PIC/s). While not a mandatory requirement of PIC/s, participating authorities may choose to recognise another’s GMP inspection, underpinned by adherence to shared standards, to alleviate burden on the pharmaceutical industry and on the resources of the respective authorities undertaking the inspections. SSA regulators could choose to unilaterally rely on the GMP inspection decisions of PIC/s members, noting these will by no means cover all manufacturing sites. Potential loss of income from GMP inspection fees may act as a disincentive, which could be overcome by maintaining a certification fee while still relieving industry of additional cost burden associated with repeat GMP inspection visits.

Achieving these goals requires the implementation of an inclusive approach across all relevant regulatory functions that encourages information exchange, harmonisation, and collaboration among and between regulatory bodies and stakeholders. Inadequacy in any regulatory function undermines the effectiveness of the process as a whole. If the authorisation function is not supported by effective inspection, pharmacovigilance or enforcement, ineffective products will remain on the market thereby undermining the regulatory process. Regional regulatory collaborations and support need to be extended to the whole supply chain.

Regulation of human medicines has been progressing significantly ahead of VMPs in Africa. One clear illustration of this is the development of the African Medicines Agency (AMA). This development intends to accelerate progress in human medicines regulation; veterinary medicines regulation improvement requires the same collective political will.

## 5 Actionable recommendations

Regulation of veterinary medicines is a complex scientific activity. In recognition of this complexity, mature veterinary medicines regulatory bodies in developed countries increasingly seek to work together to improve resilience, reduce cost, improve attractiveness of the market, and to bring medicines to the market quicker. These collaborative approaches are most developed in (but not restricted to) the EU and are underpinned by harmonisation of national operational processes. However, this collective approach to veterinary medicines regulation is least developed where it is needed most, such as, in LMICs.

Collaborative, or harmonised, approaches can improve regulatory efficiency through several different models, ranging from unilateral recognition by one country of the regulatory approvals issued by another, (particularly relevant to severely resource constrained countries), through to agreement by multiple countries to issue joint product approvals. The latter approach effectively creates a single point of access to one substantive combined market, more attractive to industry than multiple smaller national markets that each must be accessed separately. Increased similarity of national regulatory processes facilitates the operationalisation of inter-country collaboration, while familiarisation between regulators builds the trust that is essential to underpin cooperation.

There is currently a substantive variation in capability and capacity between, and within, each region of SSA. Harmonised approaches have already been initiated in each of the East, West and Southern regions, but these are at varying stages of development and there are a substantive number of countries that are not yet involved. The following recommendations would support and enhance these collaborative developments:

### 5.1 Institutional structure and capacity

Deployment of the veterinary equivalent to the WHO GBT is recommended as a supportive tool to enhance institutional capability. In addition to enabling veterinary medicine regulators to score their capability through self-assessment, the tool would enable formulation of institutional development plans and so identify priority steps for performance improvement. For maximum benefit, the tool should ultimately be hosted by an organisation with an inter-governmental mandate, such as WOAH. Furthermore, consideration should be given to support self-assessment to aid countries in applying the tool consistently, with the possibility of introducing an external verification process. Should the tool become a benchmarking tool, it can foster regulatory reliance and harmonisation, which in turn would encourage the availability of good quality medicines.

In addition to the centralised authorisation process adopted in WAEMU and planned in CEMAC countries, and the mutual recognition process adopted in EAC and progressing in southern Africa, there is an opportunity to consider ‘reliance’ as a way of managing the human and financial resource constraints faced by NRAs. Reliance is the process whereby a regulatory authority in one jurisdiction considers and gives significant weight to assessments that have been performed by another regulatory authority. A further step would be unilateral recognition and adoption of the marketing authorisation granted by another VMP regulatory body. Unfortunately, there is currently no publicly available independent assessment of VMP regulator competency, unlike the position for human medicines regulatory bodies, and until this is the case, reliance or unilateral recognition decisions would have to depend on assumed competence of VMP regulators, for example of those that are members of VICH or in the case of GMP certification, PIC/S members.

In the regional NRA collaboration approach, such as the EAC MRP, the Reference Country leads on the assessment of the full dossier which is then shared to the concerned countries for comment (32). One of the challenges that has been identified is that the Reference Countries can struggle to meet the agreed timelines. A pragmatic solution would be the sharing of evaluation work, a practice that is already in place for human medicines regulation. In this approach, NRAs each take a lead in the evaluation of specific parts of the dossier, their evaluations combined to yield a complete report which all involved NRAs then have the opportunity to review.

The advent of cloud computing has provided an opportunity to develop a cost-effective, secure, scalable and accessible IT platform to facilitate work sharing and workflow management. This platform should have the flexibility to meet the needs of mature NRAs as well as those that perform limited activities, the ability to cope with power interruptions and internet connectivity issues, and the ability to support the full range of NRA national functions, inclusive of data receipt, application tracking, inspections, pharmacovigilance, and should also support cross NRA collaboration.

There is currently no global forum where veterinary medicine regulators can discuss challenges, share intelligence and best practices and provide mutual support. Establishing an initiative to mirror the International Coalition of Medicines Regulatory Authorities (ICMRA) for human medicine regulators would provide this. Furthermore, a programme of regulatory system strengthening led by an intergovernmental body focusing on veterinary specific best practice guidelines similar to the ones developed by the WHO on good regulatory and reliance practices, would also support VMP regulation. Therefore, it is recommended that such structures are established and supported by regulators, industry, RECs, inter-governmental bodies and other stakeholders to ensure sustainability.

It is recommended that the newly established African Medicines Agency take steps to incorporate activities related to veterinary medicines regulation which would benefit the livestock and pharmaceutical sectors, improve food security, and support public health.

## 6 Conclusion

VMP regulation capacity building lags significantly behind that of human medicines. Within the veterinary medicines sector, there is a growing need and indeed interest to address existing deficiencies in veterinary drug regulation, in general and in particular within SSA. Correspondingly, initiatives have been introduced at the international or regional level aimed at harmonisation across the different countries in the region. Although challenges persist, efforts continue to be made to strengthen veterinary medicine regulation and success is starting to become apparent. This success and the pace of improvement can be enhanced by coordinated institutional resource capacity strengthening, work sharing, greater pragmatism in the routes to authorisation adopted, training, establishment of benchmarking/self-assessment and associated improvement development plans, broader engagement across the VMP regulation and supply chain, and IT infrastructure development. An improved one-health approach should be adopted by all involved in medicines regulation, especially as many of the requirements for improvement are common to human and animal medicines, and most of the benefits of improvement are mutual, this being particularly the case for control of AMR.
